# Changes in the Gut Microbiome and Pathologies in Pregnancy

**DOI:** 10.3390/ijerph19169961

**Published:** 2022-08-12

**Authors:** Kamila Gorczyca, Aleksandra Obuchowska, Żaneta Kimber-Trojnar, Magdalena Wierzchowska-Opoka, Bożena Leszczyńska-Gorzelak

**Affiliations:** Department of Obstetrics and Perinatology, Medical University of Lublin, 20-090 Lublin, Poland

**Keywords:** gut microbiota, gestational diabetes mellitus, preeclampsia, microbiome, obesity, pregnancy, fetal growth restriction, premature birth, cervical insufficiency

## Abstract

Pregnancy is a special period in a woman’s life when her organism undergoes multiple physiological changes so that the fetus has optimal conditions for growth and development. These include modifications in the composition of the microbiome that occur between the first and third trimesters of pregnancy. There is an increase in Akkermansia, Bifidobacterium, and Firmicutes, which have been associated with an increase in the need for energy storage. The growth in Proteobacteria and Actinobacteria levels has a protective effect on both the mother and the fetus via proinflammatory mechanisms. The aim of the study is to review the research on the relationship between the mother’s intestinal microbiome and gestational pathologies. Changes in the maternal gut microbiome is probably one of the mechanisms that occurs in various pregnancy diseases such as preeclampsia, fetal growth restriction, gestational diabetes mellitus, excessive gestational weight gain, and premature birth. For this reason, it seems vital to pay attention to certain interventions that can benefit the affected patients both in the short term, by preventing complications during pregnancy, and in the long term, as one of the mechanisms occurring in various gestational diseases is dysbiosis of the maternal intestinal flora.

## 1. Introduction

Pregnancy is a special time for a woman, when her organism undergoes various physiological changes so that the fetus has optimal conditions for growth and development [1,2]. These modifications pertain also to the microflora of an expectant mother. Human intestinal microbiota is currently the subject of attention of numerous researchers. Intestinal organisms and the substances they produce can be considered one of the most significant factors responsible for the health of a pregnant woman that enables the proper development of the child in the future. The human microbiota consists of approximately 100 trillion organisms that mostly inhabit the digestive tract. In the human organism, the most numerous types of bacteria inhabiting the gastrointestinal tract include Firmicutes, Bacteroidetes, Actinobacteria, and Proteobacteria. They constitute 70–90% of all bacteria in the digestive tract [3,4]. The microflora produces 3.3 million genes responsible for the production of millions of metabolites involved in the path of biochemical changes in the host [5].

The genome of microbiota is estimated to be 150 times larger than the human one [6]. The microbiome segregates food substances, such as vitamins and minerals, and carries undigested food debris further. Similar to the liver, it detoxifies and removes xenobiotics from the organism [7]. The gut microbiome is also responsible for maintaining the integrity of the gut and for the renewal of the epithelium, thereby affecting the immune system. The leakage between the proteins of the epithelium allows pathogens to enter circulation, intensifying the inflammatory reactions in the organism [6]. Increased epithelial permeability causes the penetration of bacterial lipopolysaccharides (LPSs), which has a negative effect and causes systemic inflammation, referred to as a “metabolic endotoxemia” [8]. Stimulation of the immune system takes place via toll-like 4 receptors located on the membranes of the intestinal epithelium which recognize LPSs, one of the membrane components of Gram-negative bacteria. The intestinal microbiota acts as a protective agent through many mechanisms, one of which is increasing the energy intake to enable protein synthesis by changing free fatty acids, bile acids, and LPSs to help maintain the integrity of the membranes, but the exact actions are unknown [9].

The intestinal microbiota consists of various types of bacteria, the most numerous of which are Firmicutes and Bacteroidetes, followed by Actinobacteria and Proteobacteria [10]. Diversified intestinal microbiota creates a symbiosis with itself and the host, resulting in a permanent system of non-antagonistic interactions taking part in the human metabolism [11]. Most of the world’s ecosystems have a more varied composition at the type level, whereas the gut microbiota shows a considerable variability at the species level [12]. During pregnancy, a number of metabolic, immune, and hormonal changes have an influence on the development of the fetus [13]. Throughout the time of the gestation, the gut microbiome modifies significantly to allow the fetus to develop physiologically. Estrogen and progesterone produced by the mother influence the mechanisms of regulation of the cerebral and intestinal axis and the immune activation of the intestinal mucosa [14,15]. It is assumed that the greatest inflammation occurs during implantation and childbirth compared to the third trimester of pregnancy [16]. Although the placenta produces various anti-inflammatory substances that protect the fetus, there is an inflammatory state that occurs on the surface of the intestinal mucosa and causes an increase in the amount of proinflammatory cytokines and leukocytes for the duration of the pregnancy [17]. The cooperation between the trophoblast and the immune system favors the temporal invasion of T lymphocytes, macrophages, and natural killer (NK) lymphocytes during pregnancy, leading to correct angiogenesis, participation in the transport of respiratory gases and nutrients, and protection against microorganisms [18,19,20,21]. Modifications in the composition of the microbiome occur between the first and third trimesters of pregnancy. There is an increase in Akkermansia, Bifidobacterium, and Firmicutes, which has been associated with an increase in the need for energy storage, and an increase in Proteobacteria and Actinobacteria, which, due to their proinflammatory qualities [3,4], have a protective effect on both the mother and the fetus. The maternal microbiota affects the growth of the offspring in the prenatal and postnatal period and is important in their later life [3,22].

In the first years of a child’s life, the transition of Enterobacteriaceae dominance is observed as an increase in the number of Bacteroidaceae. This indicates the maturation of the intestinal microflora, which may vary depending on the type of delivery and the infant’s diet [23,24]. The use of antibiotic prophylaxis during or immediately prior to labor is a recognized factor in reducing the number of Bifidobacterium and Bacteroides, and is associated with the risk of childhood obesity and the development of atopy [25,26,27,28]. Various studies demonstrated an increased risk of food allergy in the case of the predominance of Enterobacteriaceae over Bacteroidaceae in the intestine during infancy [29,30]. Maternal dysbiosis may permanently change the course of physiological processes, increasing the risk of certain diseases in the offspring, e.g., cardiometabolic disorders, obesity, and diabetes [31].

Taking all of this into consideration, the aim of this article is to present associations between the maternal gut microbiome and gestational pathologies.

Systematic searches were conducted in June 2022 using electronic databases such as PubMed, Science Direct, and Google Scholar in accordance with PRISMA guidelines (Moher, Liberati, Tetzlaff, Altman and Group, 2009, https://www.prisma-statement.org/ (accessed on 2 August 2022)). The databases were checked by two independent authors. The following listings were searched for: (microbiota or microbiome) AND pregnancy; intestinal microflora AND preeclampsia; gut microflora AND fetal growth restriction; intestinal microflora AND gestational diabetes mellitus; intestinal microflora AND obesity; intestinal microflora AND premature birth; gut microflora AND cervical insufficiency. The downloaded articles were first selected based on title and abstracts. As a result, we identified a total of 3125 articles related to the topic of interest. After considering the inclusion/exclusion criteria and eliminating duplicates, 87 studies were selected for analysis.

Inclusion criteria for selection in the study:

- Samples were taken from stool or placenta,
- Randomized clinical trials, systemic reviews, and meta-analyzes;
- Human research.

Exclusion criteria include:

- Case reports, conference summaries, and comments;
- Insufficient data;
- Full-text article not available for review;
- Language other than English;
- Research conducted in non-human species.

Key review references were manually searched to identify any relevant references that were missed.

## 2. Possible Beginning of the Formation of the Intestinal Microbiota in Humans

It has long been believed that the fetus develops in a sterile environment and the colonization of the gastrointestinal tract in a child takes place only during the birth and afterwards. The placenta functions as a physical and immunological obstacle between the mother and the fetus. Following the introduction of molecular sequencing as a diagnostic method, RNA was discovered in the placenta, amniotic fluid, and meconium. This evidence has been accepted by most scientists, refuting theories of sterility in fetal life [32].

In 2013, modern molecular diagnostic techniques were used to prove the presence of microbes in placental samples. This metagenomic study based on rDNA 16S revealed a typical microbiota in the placenta, including Firmicutes, Tenericutes, Proteobacteria, Bacteroidetes, and Fusobacteria, similar to the flora in the human oral cavity [33,34]. Enterobacter, Escherichia, Shigella, and Propionibacterium were found in the placenta and amniotic fluid in women after cesarean section. The Enterobacteriaceae family dominated in meconium, which indicates prenatal colonization [35].

Zheng et al. showed differences in the gut microflora between healthy-weight and macrosomic newborns. In macrosomia, the amount of Acinetobacter, Bifidobacterium, Mycobacterium, Prevotellaceae, Dyella, Bacteroidales and Romboutsia was increased [36]. Other studies have shown that the microbiota in women with HPV-positive placenta differed from women without this infection. Staphylococci and decreased Enterococacceae, Veillonellaceae, Corynebacteriaceae, and Moraxellaceae were present as compared to HPV-negative women. No response was obtained in this study as to whether there was a predisposition to the presence of pathological flora in HPV-infected patients [37].

Abrahamsson and co-authors analyzed the microbial composition of fetal and sheep intestines [38]. They showed an increase in Firmicutes and Proteobacteria in the third trimester, which may have come from contaminated reagents [39]. It is laboratory errors that are the main argument for rejecting the precision of research that shows the fetal development environment to be not sterile. Sterile sampling of the human placenta is difficult to obtain. Leiby et al. also suspected that the evidence for the existence of the placental microbiome is not scientifically reliable [40]. To be able to test it, it would be necessary to develop a method of collecting the material that does not raise ethical dilemmas and is carried out in sterile conditions, eliminating the possibility of pre-laboratory errors [41].

## 3. Gut Microbiome and Preeclampsia

Preeclampsia is a relevant issue in obstetrics, affecting 2–8% of pregnancies worldwide. It is one of the most common causes of morbidity and mortality in the perinatal period of mothers and their offspring [42,43,44,45,46]. The mechanism of preeclampsia is yet to be fully understood. There are studies in which the abnormal structure of the placenta is considered to be the cause or damage to the endothelium, and the associated vascular disease acts as a factor influencing the development of preeclampsia [47]. Preeclampsia is believed to be associated with future metabolic syndrome in which glucose and lipid metabolism is impaired, insulin resistance is present, and vascular endothelial degradation occurs [38].

The current studies describe the relationship between the maternal gut microbiota and the diagnosis of preeclampsia. In a study of 100 women, 26 were pregnant with preeclampsia, 25 had abnormal growth of the placenta, 21 were healthy non-pregnant women, and 28 were healthy pregnant women. A significant reduction in the abundance of Prevotella, Porphyromonas, Varibaculum, and Lactobacillus was observed in women with preeclampsia compared to pregnant women without this complication [40]. Prevotella is a bacterium that exerts many functions in the human gastrointestinal tract [48]. Prevotella’s use of fiber and polysaccharides in the production of short-chain fatty acids (SCFAs) such as butyrate has been proven in recent studies and publications. Various fatty acids are beneficial for the functioning of the body [49]. One of them is butyrate, which lowers the maternal blood pressure during pregnancy [50]. Butyrate is the main source of energy for cells building the intestinal epithelium, and is involved in the differentiation of T lymphocytes and affects the functioning of the immune system [49,51,52]. The presence of Prevotella in the digestive tract serves to counteract microbial infections [53].

Amarasekara et al. studied 110 pregnant individuals and confirmed the presence of bacteria in the placenta in 12.7% of women with preeclampsia, as compared with women who did not have hypertension during pregnancy [54]. This may indicate that the presence of bacteria in the placenta is a predisposing factor for the onset of preeclampsia [55].

A study by Huang et al. showed an inverse correlation between the number of Lactobacillus and the incidence of arterial hypertension in patients with preeclampsia [56]. The study also included toxins produced by Lactobacillus OTU255 and OTU784, where OTU255 was significantly reduced in the group of individuals with preeclampsia, while OTU784 decreased significantly in patients with abnormal placental growth. The analysis of the obtained results allows us to draw conclusions about the importance of changes in the microorganisms inhabiting the gastrointestinal tract in the etiology of both preeclampsia and abnormal growth of the placenta during pregnancy [40]. Lactobacillus is one of the most popular probiotics used worldwide as an additive in processed foods and drug development [56]. The above studies support the use of probiotics in pregnant women to reduce the risk of preeclampsia. The conducted studies showed a protective effect of butyrate on the occurrence of preeclampsia by inhibiting the synthesis of the plasminogen activator-1 inhibitor, which resulted in a reduction in vasoconstriction and a reduction in the secretion of nitric oxide damaging the vascular endothelium [57].

Koren et al. observed various changes on a quantitative and species level in the composition of the gut microbiota when comparing pregnant individuals in early pregnancy and in the third trimester [3]. An increase in Proteobacteria and Actinobacteria was shown. After implanting the intestinal microflora of a female in the third trimester in mice, a significant increase in the amount of fat and insulin resistance was observed. This proves the thesis that the intestinal microbiota affects the metabolism of a pregnant person [17]. A reduction in the genus Firmicutes in pregnant women with preeclampsia was shown, which includes the species: Bulleidia moorei, Clostridium perfringens, and Coprococcus catus [58]. Clostridium perfringens are involved in the metabolism of carbohydrates and proteins [59]. Living in the large intestine, they can cause intestinal disorders and gas gangrene leading to septic shock, and can affect the cardiovascular system. They can also secrete 16 different toxins [60]. The α toxin can increase blood pressure and lead to disturbances in blood coagulation, thus reducing the speed of blood transport in the body, affecting the cardiovascular system, and increasing the risk of vascular diseases [61]. The β toxin can lead to necrotizing enterocolitis, as well as the narrowing of blood vessels, which increases blood pressure [62]. The ε toxin is nephrotoxic and damages the cells of the renal tubular epithelium. Some animal studies show that this toxin can also increase blood pressure [54]. Liu et al. concluded that an increase in the amount of Clostridium perfringens may predispose a person to preeclampsia through the toxins and interactions between organisms and other microbes living in the human intestine [63]. The authors, by using 16S rDNA gene sequencing from the feces of pregnant individuals, demonstrated a decrease in the probiotic Coprococcus catus in mothers with preeclampsia in comparison to healthy pregnant women [58].

## 4. Fetal Growth Restriction

Fetal growth restriction (FGR) is a common obstetric complication and may also be known as intrauterine growth restriction (IUGR). The factors involved in the pathogenesis of FGR include: infections, maternal age, malnutrition, genetic disorders, and insufficient placenta to supply the fetus with nutrients [64]. Multiple studies suggest that the gut microbiome can also participate in the pathogenesis of FGR.

Den Hollander et al. showed a correlation between Helicobacteri pylori and the occurrence of FGR in a group of 6000 pregnant women [65]. Groer et al. concluded that the birth weight of a child is a significant factor in the balance of intestinal microbes in infants, and thus influences their further growth and development [63]. A study of 150 pairs of twins using 16S ribosomal RNA and metagenomic sequencing showed a correlation between increased bacterial diversity early in life and intrauterine FGR in twins. A reduction in Enterococcus and Acinetobacter numbers was observed in twin-born FGR infants and there was a lowering in the level of methionine and cysteine in stool samples taken after birth and after 2–3 years of follow-up [66]. It is also suspected that the level of cysteine in the stool may be correlated with the future physical development of the child [66]. Oscillospira and Coprococcus participate in the synthesis of butyrate, which is an energy source for the epithelial cells of the small intestine, regulating glucose metabolism and reducing inflammation in the organism [67]. The study by Yang’s team showed an increased number of the above-mentioned butyrate markers in twins with FGR, which may compensate for intrauterine malnutrition [66]. By sequencing the 16S rDNA amplicon collected from pregnant women with FGR and the control group, stool samples showed significant differences in the growth of Bacteroides, Faecalibacterium and Lachnospira in patients with FGR [68]. Fernandez-Gonzalez et al. are currently conducting a study on the composition of the gastrointestinal microorganisms and inflammatory relationships with a growth appropriate to the gestational age in 63 fetuses with FGR and in the control group [69].

## 5. Gestational Diabetes Mellitus

There is an increasing trend towards the occurrence of GDM worldwide, contributing to an increased risk of obesity, T2DM, and metabolic syndrome [50,70,71,72,73]. GDM is one of the most common metabolic complications of pregnancy, with an incidence ranging from 1.8% to 22% [74]. The intestinal microbiota is involved in metabolic changes that affect the blood glucose level [75]. The influence of intestinal dysbiosis on the development of GDM is a contentious issue for many scientists. Changes in various taxa are shown, including types, genera, and species, especially in mid- and late gestation [76,77]. Cortez et al. showed an increase in Firmicutes and a decrease in Bacteroidetes in GDM patients, as well as an increase in the Firmicutes/Bacteroidetes (F/B ratio) during the third trimester of pregnancy [78]. The F/B ratio is considered to be a marker of low-grade systemic inflammation in obesity and insulin resistance [79]. Furthermore, Sililas et al. observed that F/B in the third trimester of pregnancy was higher in patients with GDM compared to the control group [80].

Karamali et al. did not show an increase in the number of Lactobacillales in the second and third trimester of pregnancy in patients with GDM despite supplementation with probiotics [81]. It seems to be associated with molecular mechanisms involved in the reduction of probiotics from mid-pregnancy [82]. Despite this finding, the use of probiotics in pregnant women with GDM has many benefits, as numerous studies present. These profits include increasing insulin sensitivity, reducing inflammation in the organism, and decreasing the risk of preeclampsia and preterm birth [83,84,85,86,87]. An increase in the amount of Lactobacillales relieves the inflammation of the intestines and reduces insulin resistance [88].

Ferrocino et al. revealed an increase in Firmicutes and a reduction in Bacteroidetes and Actinobacteria in pregnant patients with GDM between 24 and 28 weeks of pregnancy in a sequencing study of 16S fecal microbiome amplification [89]. A metagenomic sequencing study performed in pregnant women at the 21–29 week of pregnancy revealed a dominance of Bacteroides and Klebsiella in the GDM group and of Methanobrevibacter smithii, Alistipes, Bifidobacterium, and Eubacterium in the control group [76].

Many researchers compared the composition of the intestinal microflora of pregnant women with GDM and normoglycemic mothers. Pregnant women with GDM were characterized by an increase in the number of microflora of Collinsella, Rothia, Desulfovibrio, Actinobacteria [90], Firmicutes [78,89], Parabacteroides distasonis, Klebsiella variicola [76], Ruminococcus, Eubacterium, and Prevotella [78], as well as a reduced number of Akkermansia, Bacteroides, Parabacteroides, Roseburia, Dialister [78], Methaniirevibacter, Alistipes, Bifidobacterium species, and Eubacterium species [76].

The molecular mechanisms by which intrauterine exposure to hyperglycemia in mothers with GDM contribute to the development of obesity and diabetes in the future lives of their offspring remain to be elucidated [91]. It is possible that altered gut microbiota in fetal programming is involved.

## 6. Overweight, Obesity, and Excessive Weight Gain in Pregnancy

Overweight and obesity affects the occurrence of metabolic and autoimmune diseases during pregnancy. Considering the fact that two-thirds of pregnant individuals exceed the recommendations for weight gain during pregnancy, excessive weight gain during pregnancy appears to also be a significant obstetric problem. Not only is overweight and obesity associated with complications in the offspring, but excessive weight gain in pregnancy is also, although the mechanisms are still unclear [92].

Studies have shown that most of the gut microflora in overweight people was made up of Bacteroides and Firmicutes [93]. The findings of Zacarías et al. showed that levels of Firmicutes, Fecal cocci, Streptococcus, and Actinomycetes were increased in obese mothers [94]. Overweight and obesity is associated with both inflammation in pregnant persons and a specific composition of their gut microflora. It has been shown that the index of inflammatory markers is higher in expectant mothers with excess body weight than in healthy pregnant individuals [95]. Research has proven that acetyl glycoproteins are associated with excess body weight, T2DM, and insulin resistance [96]. Furthermore, the increased concentrations of inflammatory markers such as haptoglobin and hsCRP have been observed in obese patients in the third trimester [94]. In pregnant patients, a decrease in the diversity of the intestinal microflora leads to an increase in haptoglobin and hsCRP [97]. An influence of the intestinal microbiota on insulin resistance and fat metabolism has been proven. A study involving 29 overweight and 41 obese pregnant women showed a correlation between microbes in the stool and metabolic changes [84]. Increased levels of Verrucomicrobia have been found, which may be related to the poor metabolic state of the organism [76].

The research of Angelakis et al. showed an increase in the number of Bacteroidetes and Firmicutes types, which includes the Lactobacillus species in obese and overweight people compared to adults with a healthy body weight [98]. In two studies, Million et al. revealed that the amount of Lactobacillus reuteri is correlated with obesity, whereas Bifidobacterium and Methanobrevibacter smithii do not correlate with excess body weight [99,100].

In a study conducted among 98 pregnant patients, the inverse correlation of Firmicutes to Bacteroidetes was obtained in obese mothers compared to the control group [101]. Furthermore, Collado et al. observed an increase in Bacteroides and Staphylococcus in overweight pregnant women [102]. On the other hand, there are also studies questioning changes in the F/B ratio in people with obesity. Duncan et al. revealed that the amount of Bacteroides did not change significantly when comparing lean and obese people [103]. Aatsinki and his team studied overweight pregnant patients at 24 weeks of gestation and showed a reduced number of Bacteroidetes and Bifidobacterium, as well as an increase in the genus Firmicutes [97]. Obesity may be associated with a reduced diversity of gut bacteria at both the genus and species levels, and the F/B ratio in obesity is increasing. The discrepancies in the presented results may be related to the different ages of the respondents, genetic and ethnic diversity, and dietary choices.

Obesity in pregnant people correlates with the specific microbial composition of the human intestine [17,89,104]. In overweight pregnant women, compared to those with a healthy body weight, the level of Bacteroides and Staphylococcus was increased during stool analysis [102]. Overweight and obese patients produce an increased amount of insulin and fatty cytokines, which affects the number of intestinal bacteria and confirms the research on the relationship between the microbiome and the index of the amount of metabolic hormones in pregnancy [105]. In pregnant patients with pre-pregnancy obesity, a decrease in the number of NK cells and a decrease in pro-angiogenic factors were demonstrated, which was associated with pregnancy failure [106]. Overweight and obesity has been shown to correlate with the amount of bacteria in the intestinal microbiota, such as Parabacteroides [107,108], Lachnospira [109], Faecalibacterium prausnitzii [110], and members of the Christensenellaceae [111], Ruminococcus [101], and Bifidobacterium families [112]. It was concluded that the amount of Lachnospira and Faecalibacterium is related to the risk of asthma [107,109,113,114]. Goodrich et al. showed a protective effect against weight gain in mice after fecal transplantation from obese people [111]. In the Japanese population, there was a correlation between the increase in Blautia and the development of maternal obesity [115].

Pre-pregnancy overweight and obesity increases the probability of obstetric complications; despite the developed molecular analysis techniques, the pathogenesis of these phenomena is still not clear [116]. It seems very likely that the maternal intestinal microflora may be of great importance in this respect.

## 7. Premature Birth and Cervical Insufficiency

Cervical failure affects 1% of all pregnancies and 8% of patients with recurrent mid-trimester losses [117,118]. Many studies have shown that pathogens in the amniotic fluid are present in 8–52% of pregnant women with cervical insufficiency [48,119,120,121,122,123]. The use of molecular diagnostic methods provided new information on the role of the gut microbiota in generating the risk of preterm labor. Shiozaki et al. observed a decrease in Clostridium and Bacteroides in 10 women with preterm delivery compared to a control group of those with term pregnancies [124]. Clostridium affects the immune system by activating regulatory T cells and Bacteroides T reg cells in the intestines, and induces interleukin 10 (IL-10) secretion with polysaccharide A, suppressing the Th-17 response [125,126,127]. An analysis of stool samples of 19 patients who gave birth prematurely and 102 who had a full-term birth showed a decrease in Bifidobacterium and Streptococcus, and in Clostridiales families [128]. Bifidobacterium strains show anti-inflammatory properties through the production of IL-8 [129,130]. Reducing the amount of Bifidobacterium may lead to an increased susceptibility to preterm labor in women.

## 8. Conclusions

Pregnancy can be regarded as a special period in the life of a woman, inducing multiple transformations in various organs and systems, including modifications to the immune system [131]. One of the mechanisms that occurs in various pregnancy diseases mentioned above is dysbiosis of the maternal intestinal flora. Table 1 summarizes the results of multiple studies on changes in the gut microbiota depending on the disease developing during pregnancy.

It can be argued that various complications arising during pregnancy may predispose mothers or their offspring to develop lifestyle diseases later in life, including type 2 diabetes, cardiovascular disease, obesity, and metabolic syndrome. Thus, it seems vital to scrutinize certain interventions benefiting the patients not only in the short term—by preventing complications during pregnancy—but also in the long term. A number of diseases such as GDM and preeclampsia can affect the future of the mother and her offspring as they participate in maternal and fetal programming.

Further research on the biomarkers of inflammation should be conducted, as they may constitute an important tool for the assessment of pregnancy complications, as well as for the introduction to early prevention of multiple metabolic and immune disorders. We are waiting for the results of research which, by using cutting-edge and costly methods, will bring us closer to discovering the impact of the intestinal microflora on various complications observed during pregnancy.

## Figures and Tables

**Table 1:** Changes in the intestinal microflora and gestational pathologies.

Complications	An Increase	A Decrease	References
Preeclampsia		Prevotella Porphyromonas Varibaculum Lactobacillusdata	[40]
	Proteobacteria Actinobacteria		[17]
	Bulleidia Moorei Clostridium perfringens Coprococcus catus data		[58]
FGR		Enterococcus Acinetobacter	[66]
	Oscillospira Coprococcus		[67]
	Bacteroides Faecalibacterium Lachnospira		[68]
GDM	Firmicutes	Bacteroidetes	[89]
		Actinobacteria	[87]
	Klebsiella Parabacteroides distasonis Klebsiella variicola	Methanobrevibacter smithii Alistipes Bifidobacterium Eubacterium Metaniirevibacter Alistipes Bifidobacterium Eubacterium	[76]
	Bacteroides Firmicutes Ruminococcus Eubacterium Prevotella	Akkermansia Bacteroides Parabacteroides Roseburia Dialister	[78]
	Collinsella Rothia Desulfovibrio Actinobacteria		[90]
Overweight, obesity, and excessive weight gain in pregnancy	Bacteroides Firmicutes		[93]
	Firmicutes Fecal cocci Streptococcus Actinomycetes		[94]
	Verrucomicrobia		[76]
	Bacteroidetes Firmicutes Lactobacillus species		[98]
	Lactobacillus reuteri		[99,100]
	Firmicutes Bacteroidetes		[101]
	Bacteroides Staphylococcus		[102]
	Firmicutes	Bacteroidetes Bifidobacterium	[97]
	Parabacteroides		[107,108]
	Lachnospira		[109]
		Faecalibacterium prausnitzii	[110]
		Christensenellaceae	[111]
		Ruminococcus	[101]
		Bifidobacterium families	[119]
	Blautia		[115]
Premature birth and cervical insufficiency		Clostridium Bacteroides	[124]
		Bifidobacterium Streptococcus Clostridiales	[128]

GDM (gestational diabetes mellitus); FGR (fetal growth restriction).

## Data Availability

Not applicable.
